# Development, evaluation and validation of a screening tool for late onset bacteremia in neonates – a pilot study

**DOI:** 10.1186/s12887-019-1633-1

**Published:** 2019-07-24

**Authors:** Sandra A. N. Walker, Melanie Cormier, Marion Elligsen, Julie Choudhury, Asaph Rolnitsky, Carla Findlater, Dolores Iaboni

**Affiliations:** 1 Department of Pharmacy E-302, Sunnybrook Health Sciences Centre (SHSC), 2075 Bayview Avenue, Toronto, ON M4N 3M5 Canada; 2 Leslie Dan Faculty of Pharmacy, University of Toronto, Toronto, Ontario Canada; 3 SHSC, Women and Babies Program, Toronto, Ontario Canada

**Keywords:** Neonates, Late onset bacteremia, Screening tool

## Abstract

**Background:**

Clinical and laboratory parameters can aid in the early identification of neonates at risk for bacteremia before clinical deterioration occurs. However, current prediction models have poor diagnostic capabilities. The objective of this study was to develop, evaluate and validate a screening tool for late onset (> 72 h post admission) neonatal bacteremia using common laboratory and clinical parameters; and determine its predictive value in the identification of bacteremia.

**Methods:**

A retrospective chart review of neonates admitted to a neonatal intensive care unit (NICU) between March 1, 2012 and January 14, 2015 and a prospective evaluation of all neonates admitted between January 15, 2015 and March 30, 2015 were completed. Neonates with late-onset bacteremia (> 72 h after NICU admission) were eligible for inclusion in the bacteremic cohort. Bacteremic patients were matched to non-infected controls on several demographic parameters. A Pearson’s Correlation matrix was completed to identify independent variables significantly associated with infection (*p* < 0.05, univariate analysis). Significant parameters were analyzed using iterative binary logistic regression to identify the simplest significant model (*p* < 0.05). The predictive value of the model was assessed and the optimal probability cut-off for bacteremia was determined using a Receiver Operating Characteristic curve.

**Results:**

Maximum blood glucose, heart rate, neutrophils and bands were identified as the best predictors of bacteremia in a significant binary logistic regression model. The model’s sensitivity, specificity and accuracy were 90, 80 and 85%, respectively, with a false positive rate of 20% and a false negative rate of 9.7%. At the study bacteremia prevalence rate of 51%, the positive predictive value, negative predictive value and negative post-test probability were 82, 89 and 11%, respectively.

**Conclusion:**

The model developed in the current study is superior to currently published neonatal bacteremia screening tools. Validation of the tool in a historic data set of neonates from our institution will be completed.

**Electronic supplementary material:**

The online version of this article (10.1186/s12887-019-1633-1) contains supplementary material, which is available to authorized users.

## Background

The increased risk of late-onset infections (greater than 72 h following birth) in preterm and very low birth weight (VLBW) neonates is well documented [1]. Despite advancements in care, late-onset sepsis occurs in up to 20% of VLBW infants, with 28% of septic neonates experiencing more than one episode [2].

The diagnosis of late-onset neonatal sepsis is reached using various signs and symptoms, and often leads to the initiation of empiric, broad spectrum antimicrobial therapy before laboratory results are available [2]. In a study by Wirschafter et al., it was found that the ratio of antibiotic courses administered to the number of confirmed blood stream infections (BSIs) in neonates was 14:1, suggesting that antibiotic overuse is an issue that needs to be addressed in this patient population [3].

The reason for antibiotic overuse in the neonatal population is multifactorial. The lack of specificity of symptoms of bacteremia and the overlap of shared symptoms among various neonatal conditions produces an extensive list of differential diagnoses for clinicians to consider and may lead to the overuse of broad-spectrum antibiotics. Because the sensitivity of laboratory diagnosis of BSIs in neonates is affected by the small volumes of blood permissible in blood draws (0.5 mL), clinicians cannot rely on blood cultures alone, with false negative rates of up to 60% in low colony count sepsis [4]. Currently, healthcare professionals in the neonatal intensive care unit (NICU) lack a standardized, validated prediction tool for bacteremia. Published screening tools that predict bacteremia have deficiencies in their performance metrics (e.g. sensitivity and specificity) which limit their application in clinical practice [5–14].

In addition to common clinical [15] and laboratory parameters that are used to subjectively predict bacteremia and sepsis in neonates, acute phase reactants such as C-reactive protein (CRP) and procalcitonin (PCT) [16, 17] are being investigated; however, they have limitations [17] and are either not routinely measured or quickly available in most hospitals. Similarly, although the intercellular messenger CD64 has been shown to be an accurate diagnostic marker of early- and late-onset neonatal sepsis [18], it is not routinely measured in clinical practice. Other novel predictors of infection have also surfaced [16, 19–23], and although the investigation of these new biomarkers as predictors of neonatal sepsis is exciting and may be promising in the future, they are unavailable to clinicians today.

Given the rate of antibiotic use in the NICU, a practical screening tool for bacteremia would enable safer, more appropriate use of antibiotics. An ideal screening tool for bacteremia in neonates should provide sufficient sensitivity to ensure a case of bacteremia is not missed, with a low negative post-test probability so as to promote a decrease in empiric, broad spectrum antimicrobials in non-bacteremic neonates. The objective of this study was to develop, evaluate and validate a screening tool for late onset (> 72 h post admission) neonatal bacteremia using common laboratory and clinical parameters.

## Methods

### Study design

This pilot study was approved with the need for informed consent waived by the Sunnybrook Health Sciences Centre (SHSC) Research Ethics Board on January 13, 2015. The study employed a prospective and retrospective study design. The retrospective cohort of neonates included all eligible patients admitted to the institution’s 48 bed level 3 NICU from March 1, 2012 to January 14, 2015. The prospective cohort of neonates were all eligible patients admitted to the institution’s NICU from January 15, 2015 to April 30, 2015.

All neonates admitted to the institution’s NICU during the study period were eligible for study inclusion, regardless of gestational age. Neonates that did not have at least some relevant laboratory parameters or vital signs collected during their stay were excluded, as they did not have data to contribute to the development of the screening tool. This included neonates admitted to the NICU for hyperbilirubinemia and hypoglycemia (unrelated to sepsis) who only had laboratory monitoring of bilirubin and/or blood glucose, as well as neonates staying in the NICU for less than 48 h who did not have laboratory parameters or vital signs collected, recorded, or accessible to the team collecting data. Only neonates with late-onset bacteremia (bacteremia occurring greater than 72 h after admission to the NICU) were eligible for study inclusion in the case cohort.

### Data collection

Data on 35 clinical parameters and 17 laboratory parameters were collected for bacteremic cases and controls (retrospective and prospective cohorts) (Additional file 1: Table S1). These parameters were selected based on previously established and hypothesized potential signs and symptoms of bacteremia in neonates. Data for the retrospective component of the study were obtained from archived charts in the SHSC Health Records Office, the Electronic Patient Record (EPR), and the antimicrobial stewardship database.

For the prospective component, the clinical and laboratory parameter data were collected daily by a team of NICU pharmacists for all patients included in the study from date of NICU admission (day 0) to the date of first positive blood culture, discharge from the NICU, or death (whichever came first).

Following data collection, neonates with documented bacteremia (cases) were matched to non-infected neonates (controls) to reduce the risk of differences in baseline characteristics with univariate analysis having some unknown confounding effect on parameters that may influence the diagnosis of bacteremia. The identification of significant parameters with univariate analysis and subsequent confirmation with Pearson’s correlation provided justification of parameter entry into the binary logistic regression. At the point of data entry into the binary logistic regression there is no further importance related to matching; and therefore, relevant data from both matched and unmatched controls were eligible for tool development using binary logistic regression to maximize sample size. Control patients were neonates who did not receive antibiotics during their NICU hospital admission beyond the first 48 h of life and never had a positive culture at any site. Cases were matched to controls based on gender (when possible), gestational age at birth, corrected gestational age at study entry, weight at study entry, total length of stay in the NICU, and antibiotic use (yes or no) within the first 48 h of life. The remainder of control patients were categorized as unmatched controls.

Neonates were categorized as having late-onset bacteremia if a blood culture was positive for non-contaminant bacteria more than 72 h into their NICU admission. Neonates with blood isolates considered to represent contaminants (*Corynebacterium* spp., *Propionibacterium* spp., and *Bacillus* species other than *B. anthracis* [24]) were excluded from further comparative analysis of bacteremic versus non-bacteremic patients to avoid any potential confounding. The criteria for a true coagulase negative *Staphylococcus* spp. (CONS) infection in neonates varies [25, 26], therefore for the purpose of the current study, neonates found to have blood cultures positive for CONS were included as bacteremic cases for analysis if the colony count was reported as greater than 100 colonies or if appropriate antibiotics were used for 7 or more days in response to the positive culture and correlated clinical status of the patient. If the colony count for CONS was less than 10 colonies or antibiotics were used for less than 7 days in response to the positive culture, the neonate was excluded from analysis.

At the time of their first positive non-contaminant blood culture, neonates were classified as cases and matched one-to-one to controls for analysis. The time of the positive blood culture represented the time of study entry for bacteremic cases. In the event that a patient had multiple positive blood cultures during their NICU hospital stay, data was only collected in relation to their first positive blood culture identified > 72 h into their NICU admission.

The data collected for final analyses were the parameter results closest to but before the date of blood culture collection within the previous 24 h period in cases and the variable result closest to the matching length of stay day post-birth within the previous 24 h period for controls (i.e. if case patient had positive blood culture 96 h after birth, then relevant parameters for case and their matched control patient were obtained from 72 h to 96 h after birth). In the case of laboratory parameters that were infrequently ordered (CRP and lactate), the respective closest value within a period of 96 h before the blood culture collection date (in cases) or matched days post-birth (in controls) was recorded. In the case of clinical parameters in which a maximum or minimum value was needed, the parameters were defined as being the maximum or minimum within 24 h before the date of blood culture collection in cases or matched days post birth in controls. Data on unmatched controls were obtained from the neonate’s worst day in the NICU using fraction of inspired oxygen (FiO2) as the marker given the highest priority for determining worst NICU day. For neonates who were not ventilated and on room air, the worst NICU day was the day with the most out of range clinical or laboratory parameters.

### Data analysis

#### Sample size

In the literature, there is currently no standard ratio to determine how many patients are required per independent variable analyzed in the development of a screening tool. Traditionally, minimum ratios from 2:1 to 10:1 (patients to variables), and a minimum sample size of 100–200 patients has been considered acceptable [27–32]. A target sample size of 100 neonates would allow for assessment of a maximum of 10 (at a ratio of 10:1) up to 50 (at a ratio of 2:1) variables for association with bacteremia in the evaluation to create a screening tool. A total of 52 clinical and laboratory parameters were included for potential assessment in the current study. If each of these parameters was significant with univariate analysis and entered into the iterative binary logistic regression modelling, a minimal sample size of 104 neonates (for a ratio of 2 patients to 1 variable) would be required.

#### Statistics

Descriptive statistics (mean with standard deviation or median, and range or percentage) were used to describe patient characteristics. Univariate analyses using a two-tailed unpaired t-test (interval data normally distributed), two-tailed unpaired t-test with Welch correction for normally distributed data with unequal standard deviations; Mann-Whitney U test (interval data not normally distributed, or ordinal data), or Fisher’s Exact Test and odds ratios with 95% confidence interval (nominal data) (GraphPad Instat, version 3.05, 32 bit for WIN 95/NT, created September 27, 2000) were used to compare patient characteristics, clinical parameters, and laboratory values obtained from cases versus controls. One-way analysis of variance (ANOVA) (interval parametric data) and Kruskal-Wallis (interval nonparametric data) were used when comparing characteristics across > 2 groups of patients. A Pearson’s Correlation matrix (SPSS version 13.0 for Windows, created September 1, 2004) was completed to identify clinical and laboratory parameters (independent variables) associated with bacteremia (dependent variable) (thereby, confirming the univariate analyses) and to determine the percentage of patients with a given measured variable. Any clinical and laboratory parameters available for > 20% of patients and having a *p* value < 0.05 with both univariate analysis and Pearson’s Correlation were entered into binary logistic regression (SPSS version 13.0 for Windows, created September 1, 2004) using an iterative process to identify a statistically significant model (*p* < 0.05) in which all independent variables remaining in the model had an odds ratio of > 1 and which provided the highest sensitivity and specificity. Only patients with a complete data set for the identified significant independent variables were included in the development of the final model. A Receiver Operating Characteristic (ROC) curve was developed to identify the optimal probability breakpoint representing bacteremia. Classification and Regression Tree Analysis (CART) (Salford Predictive Modeler 7.0 Pro 32mb) was used to identify breakpoints of each independent variable that remained significant in the final model. Sensitivity and specificity analysis was conducted on the best predictive model for bacteremia. The optimal bacteremia screening tool developed was compared to published tools by mapping the sensitivity and false positive rate (1-specificity) for all tools to generate a ROC curve.

## Results

**Fig. 1:**
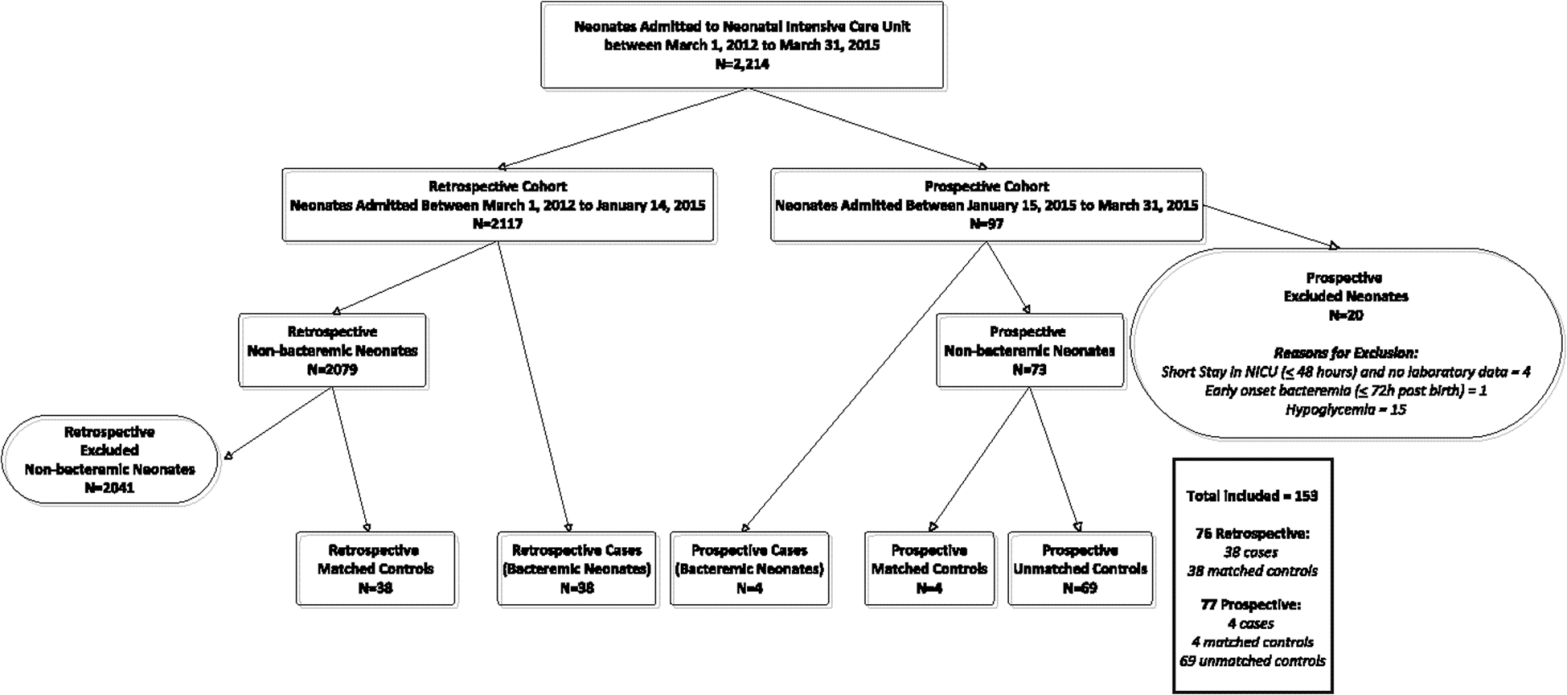
Patient Eligibility Flow Chart

A total of 2214 neonates were admitted to the NICU between March 1, 2012 – March 31, 2015 and 153 of these neonates (7%) (42 cases, 42 matched controls, 69 prospective unmatched controls, 111 total controls) were included in this study (Fig. 1). Patient characteristics of the entire study population (*n* = 153) and patient characteristics of the sample of patients that had a complete data set for inclusion in the development of the final bacteremia screening tool (cases = 31, controls = 30) are detailed in Additional file 1: Tables S2 and S3, respectively*.* The overall period prevalence of bacteremia at the study hospital during the study period was 2% (42/2214). Six of 111 control patients (5%) (including 3 matched control patients (3/42, 7%)) had blood cultures drawn and processed, each of which was negative for any microbial growth. One of these control patients had complete data and was included in tool development (1/30, 3%). The majority of organisms isolated in blood samples for bacteremic cases were Gram Positive bacteria (38 out of 45 isolates, 84%) (Additional file 1: Table S4).

The 26 parameters found to be significantly correlated with bacteremia by univariate analysis are detailed in Additional file 1: Table S5. Significant parameters that were identified in univariate analysis, but were not input into the iterative binary logistic regression process were: mortality that was possibly related to bacteremia, survival at the end of NICU stay, number of days in NICU and number of ventilation days, since these would not be parameters known to a clinician at the time of using the screening tool in clinical practice, and therefore would not be helpful in a predictive tool; maximum mean arterial pressure (MAP) was excluded because there is no normal range in babies and it is influenced by corrected gestational age; all parameters with a significant negative correlation (gestational age at birth, corrected gestational age at entry, weight at entry, minimum temperature, and maximum serum creatinine) were excluded because they would not be helpful in a predictive tool to identify bacteremia. Therefore, of the original 26 significant parameters identified by univariate analysis, only 16 parameters were assessed in the iterative binary logistic regression. Sixty-one neonates had a complete data set for inclusion in the development of the optimal binary logistic regression model (31 cases, 30 controls). Therefore, the patient to variable ratio for the iterative binary logistic regression process was 4:1, which is considered acceptable [27–32]. Of the cases included in the final data set for tool development, 29 were from the retrospective chart review and 2 were from the prospective chart review. Of the controls included in the final data set for tool development, 2 were matched controls from the retrospective chart review and 28 were unmatched controls from the prospective chart review. The remaining neonates with missing clinical and/or laboratory values were excluded (*n* = 92; 10 cases, 82 controls).

The optimal binary logistic regression model for the bacteremia screening tool (Table 1) was **Ln (odds of bacteremia)** = − 25.459 + 0.752(Maximum Blood Glucose [mmol/L]) + 0.119(Maximum Heart Rate [bpm]) + 0.108(% Bands) + 0.071(Maximum Neutrophils [× 10 〈9〉/L]).

**Table 1:** Optimal model for Bacteremia in neonates

Binary logistic regression analysis (significant model *p* < 0.0001, Nagelkerke Correlation Coefficient 66%; *N* = 61 patients with a complete data set)			
Ln (Odds Bacteremia (*Y* / *N*)) = − 25.459 + 0.752(Maximum Blood Glucose [mmol/L]) + 0.119(Maximum Heart Rate [bpm]) + 0.108(% Bands) + 0.071(Maximum Neutrophils [× 10^9^/L])			
Variables in Final Binary Logistic Regression Equation			
Independent Variable	Odds Ratio	95% Confidence Interval	CART breakpoint for association with bacteremia when parent node is maximum blood glucose
Maximum Blood Glucose (mmol/L)	2.121	1.182–3.806	> 6
Maximum Heart Rate (bpm)	1.127	1.040–1.221	> 186
% Bands	1.114	0.574–2.160	> 2.15
Maximum Neutrophils (× 10^9^/L)	1.073	0.932–1.236	> 11.7

Therefore, odds of bacteremia is the exponential of the preceding equation and the probability of bacteremia = Odds of Bacteremia/ (1 + Odds of Bacteremia). Using a ROC curve, the optimal probability cut-off for bacteremia (i.e. the threshold above which a neonate would be deemed to be bacteremic) was found to be > 41.5% with an area under the curve of 89%. The CART determined breakpoints for the parameters in the bacteremia screening tool are detailed in Table 1.

**Fig. 2:**
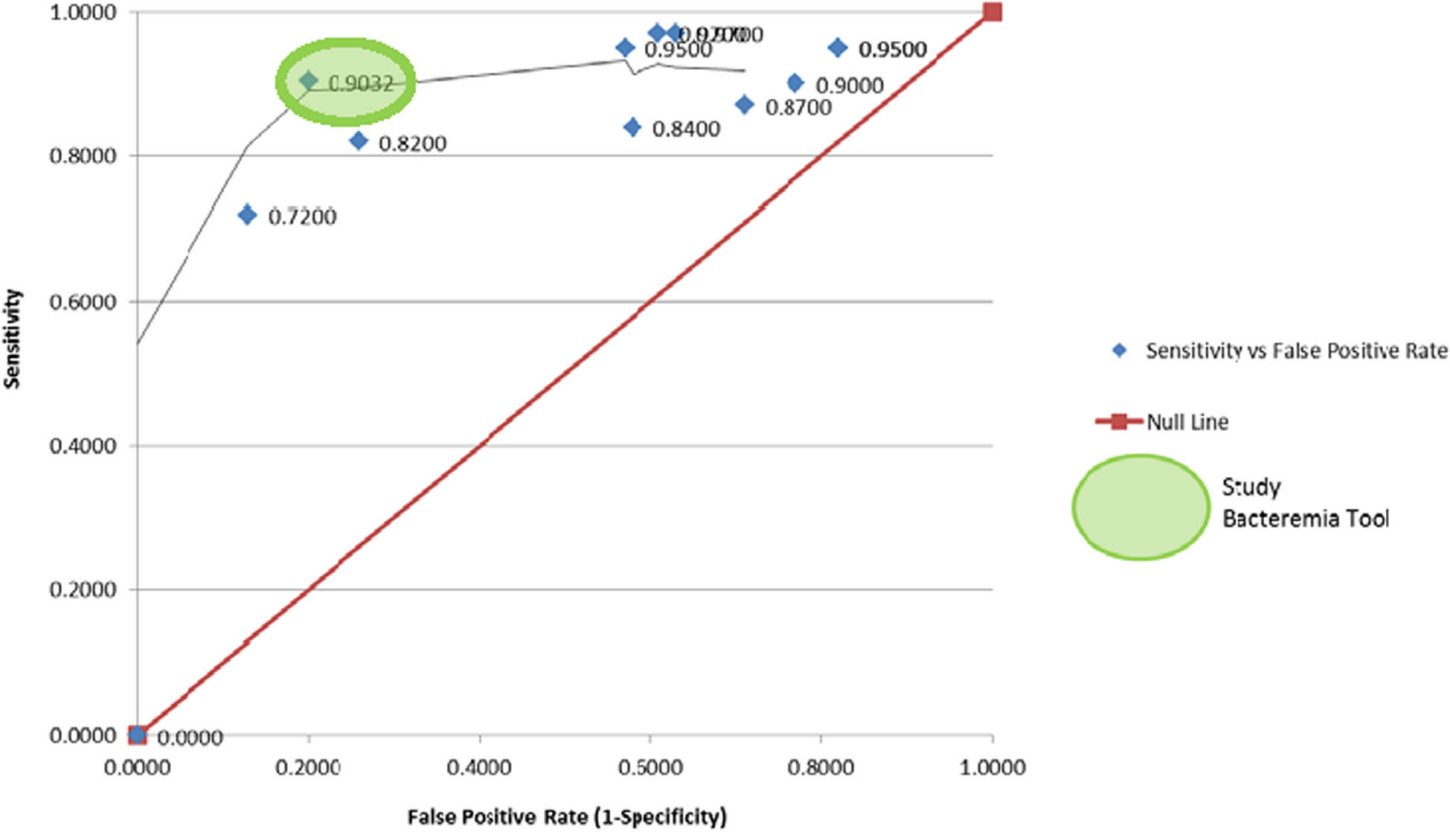
Receiver Operating Characteristic Curve Comparing Study Bacteremia Screening Tool to Currently Published Screening Tools [5–14]

The optimal model has a sensitivity of 90% (false negative rate of 10%), a specificity of 80% (false positive rate of 20%), and an overall accuracy of 85%. Positive and negative likelihood ratios were 4.50 and 0.12 respectively. The screening tool’s positive predictive value (PPV) was 82%, and the negative predictive value (NPV) was 89%. At the study population’s pre-test probability of 51%, the screening tool had a negative post-test probability of 11%. At the overall study period prevalence of bacteremia of 2%, this translates to a negative post-test probability of 0.2% (Additional file 1: Table S6). Importantly, Additional file 1: Table S6 could be used by clinicians and investigators: i) to identify the predicted PPV, NPV, and negative post-test probability of our tool at the bacteremia prevalence (pre-test probability) in their hospital and ii) to compare our tool to other published tools reporting a different bacteremia prevalence. When compared to other screening tools using a ROC curve, our model had the lowest false-positive rate while maintaining a high sensitivity (Fig. 2 and Table 2).

**Table 2:** Performance comparison of study developed tool to currently published screening tools [5–14]

	Sensitivity	Specificity	Period Prevalence	Positive Post-Test Probability	Negative Post-Test Probability	Positive Likelihood Ratio	Negative Likelihood Ratio	False Positive rate	False Negative Rate	Study Developed Tool Negative Post-Test Probability at Citation Bacteremia Period Prevalence
Study Developed Tool										
*P* value > 0.415	0.90	0.80	0.51	0.82	0.11	4.50	0.12	0.20	0.10	–
Mahieu et al., 2000 [7]										
Score ≥ 8	0.95	0.43	0.41	0.54	0.08	1.67	0.12	0.57	0.05	0.08
Score ≥ 11	0.60	0.84	0.41	0.72	0.25	3.75	0.48	0.16	0.40	0.08
Score ≥ 14	0.26	1.00	0.41	1.00	0.34	9999.00	0.74	0.00	0.74	0.08
Score ≥ 11 plus positive culture	0.72	0.87	0.41	0.79	0.18	5.50	0.32	0.13	0.28	0.08
Mahieu et al., 2002 [5]										
Score ≥ 11	0.84	0.42	0.55	0.64	0.32	1.45	0.38	0.58	0.16	0.13
Score ≥ 11 + 3 RFs	0.82	0.67	0.55	0.75	0.25	2.48	0.27	0.33	0.18	0.13
Singh et al., 2003 [8]										
Score ≥ 1	0.87	0.29	0.29	0.33	0.16	1.23	0.45	0.71	0.13	0.05
Score ≥ 2	0.53	0.80	0.29	0.52	0.19	2.65	0.59	0.20	0.47	0.05
Okascharoen et al., 2005 [9]										
Score ≥ 4	0.82	0.74	0.17	0.39	0.05	3.15	0.24	0.26	0.18	0.02
Score ≥ 5	0.70	0.82	0.17	0.44	0.07	3.89	0.37	0.18	0.30	0.02
Score ≥ 6	0.47	0.96	0.17	0.71	0.10	12.00	0.55	0.04	0.53	0.02
Dalgic et al., 2006 [10]										
Score = 6–12	0.56	0.71	0.39	0.55	0.28	1.93	0.62	0.29	0.44	0.07
Okascharoen et al., 2007 [6]										
Validation Cohort Score ≤ 3 (low risk of sepsis)	0.97	0.39	0.33	0.43	0.40	1.6	0.07	0.61	0.03	0.06
Validation Cohort Score 4–7 (medium risk of sepsis)	0.77	0.43	0.33	0.48	0.27	1.35	0.53	0.57	0.23	0.06
Validation Cohort Score ≥ 8 (high risk of sepsis)	0.2	0.98	0.33	0.99	0.85	10	0.82	0.02	0.8	0.06
Kudawla et al., 2008 [11]										
≥1 clinical signs	0.90	0.23	0.27	0.30	0.14	1.17	0.43	0.77	0.10	0.04
≥2 clinical signs	0.52	0.65	0.27	0.36	0.21	1.49	0.74	0.35	0.48	0.04
≥2 markers	0.48	0.70	0.27	0.37	0.21	1.60	0.74	0.30	0.52	0.04
≥1 clinical sign + ≥ 2 markers	0.95	0.18	0.27	0.30	0.09	1.16	0.28	0.82	0.05	0.04
Rosenberg et al., 2010 [12]										
Score ≥ 1	0.77	0.50	0.54	0.64	0.35	1.54	0.46	0.50	0.23	0.12
Score ≥ 2	0.42	0.82	0.54	0.73	0.45	2.33	0.71	0.18	0.58	0.12
Bekhof et al., 2013 [13]										
1 of 4 signs present	0.97	0.37	0.27	0.36	0.03	1.54	0.08	0.63	0.03	0.04

The screening tool developed in this pilot study was validated in a small separate retrospective cohort of neonates admitted to the NICU between September 12, 2010 and February 29th, 2012 with a full data set for the tool parameters (unpublished data) (*n* = 8; bacteremic neonates, *n* = 7; non-bacteremic neonates, *n* = 1) (Additional file 1: Table S7). The tool identified all 7 bacteremic neonates and differentiated the non-bacteremic neonate from the group.

## Discussion

A screening tool that accurately predicts the probability of late-onset bacteremia in neonates using four parameters (blood glucose, heart rate, bands, and neutrophils) that are readily available through routine blood work and monitoring in the NICU was developed. In the developmental cohort, the tool has a sensitivity of 90% (false negative rate of 10%), a specificity of 80% (false positive rate of 20%), an accuracy of 85%, a positive and negative likelihood ratio were 4.50 and 0.12 respectively, a positive predictive value of 82%, a negative predictive value of 89%, and at the study population’s pre-test probability of 51%, the screening tool had a negative post-test probability of 11%. At the overall hospital study period prevalence of bacteremia during the study period of 2%, this translates to a negative post-test probability of 0.2%, meaning that the risk of missing a neonate with true bacteremia is < 1% at the study bacteremia prevalence. A user-friendly app can be accessed at https://sunnybrook.ca/content/?page=antimicrobial-stewardship-blood-screening-neo and is available at no charge for clinical use to provide clinicians with a fast calculation of the probability of BSI (%) in their patients and make recommendations for obtaining blood cultures and consideration of empiric antimicrobial management based on practical probability cut-offs (Additional file 2: Figure S1).

The tool developed in this study had the lowest false positive rate while maintaining a high sensitivity (Fig. 2) compared to previously published tools [5–14]. In addition, when an equal period prevalence was used to compare the tools, our study tool had a negative post-test probability that was equal to or lower than previously published screening tools with better overall metrics for sensitivity and specificity [5–14] (Table 2). Mahieu et al. in 2000, developed a screening tool with high sensitivity and low negative post-test probability that assigns points if various clinical and laboratory parameters, including CRP, polymorphonuclear neutrophil (PMN) fraction, temperature, number of days of Total Parenteral Nutrition (TPN), and platelet count, exceed a certain threshold [7]. The model’s performance was tested at various cut-off points, with a score of 8 or greater having the highest sensitivity and lowest negative post-test probability. Despite the screening tool’s excellent sensitivity, its ability to differentiate between bacteremic and non-bacteremic neonates is poor, with a specificity of only 43% [7] .

Despite the high sensitivity of some previously developed screening tools [5–8, 11, 13], their low specificity would result in an inability to differentiate between bacteremic and non-bacteremic neonates. While the priority is to detect all neonates with bacteremia, a tool that over-selects for bacteremia is of little use clinically.

Our study was not without limitations. Given that a portion of our study was retrospective, there is a potential for confounding factors to impact outcomes; however, we hope that the incorporation of a prospective component has minimized any confounding. Furthermore, we were unable to collect complete data sets for all neonates due to the observatory nature of the study design. The inability to collect complete data sets may have impacted on our ability to evaluate parameters which were not often obtained (e.g. change in level of consciousness, liver function tests, arterial lactate, venous lactate, and albumin). Since we only included neonates with full data sets in the final analysis to create our model, our final sample size was reduced from 153 to 61, which may have influenced our ability to identify significant parameters with Pearson’s correlation (univariate analysis) and the model development with iterative binary logistic regression. Lastly, neonates with a positive blood culture growing CONS, bacteria typically considered to be contaminants when isolated in the adult population, were included or excluded from the study based on a combination of culture result and clinical judgement. The partially subjective nature of this approach to inclusion or exclusion of a neonate from the study, although not ideal, is difficult to avoid even in a purely prospective study in neonates, due to the subjective current approach to treatment of CONS bacteremia in neonates [25, 26].

## Conclusions

A clinical tool that can be used at the bedside to determine the probability that a neonate has late-onset bacteremia could assist clinicians in the decision-making process when it comes to requesting blood cultures and initiating broad-spectrum antibiotics in the NICU. The screening tool developed in this study incorporates four parameters that are readily available to clinicians through routine monitoring and standard care. Whereas current screening tools aim only to detect bacteremia, our tool has the potential capacity to differentiate between bacteremic and non-bacteremic neonates – a feature that could be of significant value to clinicians who are deciding whether to draw blood cultures or initiate broad spectrum antibiotics in the event of negative blood cultures. While the results of the preliminary validation of our tool in a small retrospective sample of neonates were encouraging, prospective validation of the screening tool in a larger sample size is required and is planned at the study site.

## Additional files

Additional file 1 : **Table S1.** Clinical and Laboratory Data Collection Parameters. **Table S2.** Patient Characteristics of Entire Study Population (*N* = 153). **Table S3.** Characteristics of Patients Included in Final Bacteremia Tool. **Table S4.** Microbiological Characteristics in Blood Cultures. **Table S5.** Parameters Significantly Associated with Bacteremia (Univariate Analysis). **Table S6.** What Would Happen with Bacteremia Tool if Pre-Test Probability were Different?. **Table S7.** Patient Characteristics of Validation Cohort (*N* = 8). (DOCX 47 kb)

Additional file 2 : **Figure S1.** Screening tool for early identification of bloodstream infection in neonates. This figure provides a screenshot of the screening tool. (TIF 887 kb)

## Data Availability

The datasets used and/or analysed during the current study are available from the corresponding author on reasonable request.

## Abbreviations

ANOVA: Analysis of variance
BSI(s): Blood stream infection(s)
CART: Classification and Regression Tree
CONS: Coagulase negative *Staphylococcus* spp
CRP: C-reactive protein
EPR: Electronic Patient Record
FiO2: Friction of inspired oxygen
MAP: Mean arterial pressure
NICU: Neonatal intensive care unit
NPV: Negative predictive value
PCT: Procalcitonin
PMN: Polymorphonuclear neutrophil
PPV: Positive predictive value
ROC: Receiver Operating Characteristic
SHSC: Sunnybrook Health Sciences Centre
TPN: Total Parenteral Nutrition
VLBW: Very low birth weight
