# Supplementary material for: Development, evaluation and validation of a screening tool for late onset bacteremia in neonates – a pilot study
Source: BMC Pediatr. 2019 Jul 24;19:253. doi: 10.1186/s12887-019-1633-1 (PMC6651932; doi:10.1186/s12887-019-1633-1)
Supplement: Supplementary file 1 — : Table S1. Clinical and Laboratory Data Collection Parameters. Table S2. Patient Characteristics of Entire Study Population (N = 153). Table S3. Characteristics of Patients Included in Final Bacteremia Tool. Table S4. Microbiological Characteristics in Blood Cultures. Table S5. Parameters Significantly Associated with Bacteremia (Univariate Analysis). Table S6. What Would Happen with Bacteremia Tool if Pre-Test Probability were Different?. Table S7. Patient Characteristics of Validation Cohort (N = 8). (DOCX 47 kb) [file 12887_2019_1633_MOESM1_ESM.docx]

**Additional file 1 for:**

**Development, Evaluation and Validation of a Screening Tool**

**for Late Onset Bacteremia in Neonates – A Pilot Study**

Sandra A.N. Walker^1,2,3,4*^, PharmD; Melanie Cormier^1^, BScPhm; Marion Elligsen^1^, BScPhm; Julie Choudhury^5^, PharmD; Asaph Rolnitsky^5^, MD; Carla Findlater^5^, PharmD; Dolores Iaboni^5^, BScPhm

^1^Sunnybrook Health Sciences Centre (SHSC), Department of Pharmacy, Toronto, Ontario ^2^University of Toronto, Leslie Dan Faculty of Pharmacy, Toronto, Ontario

^3^SHSC, Division of Infectious Diseases, Toronto, Ontario

^4^SHSC, Sunnybrook Research Institute, Toronto, Ontario, Canada

^5^SHSC, Women and Babies Program, Toronto, Ontario, Canada

***Corresponding:**

Dr. Sandra A.N. Walker

Department of Pharmacy E-302

Sunnybrook Health Sciences Centre

2075 Bayview Avenue

Toronto, ON

M4N 3M5

Email address: [sandra.walker@sunnybrook.ca](mailto:sandra.walker@sunnybrook.ca)

Tel: 416-480-4494 Fax: 416-480-5877

**Table S1. Clinical and Laboratory Data Collection Parameters**

| **Clinical Parameters** | **Laboratory Parameters** |
| --- | --- |
| Gender | White blood cell count (maximum) |
| Gestational age at birth | Polymorphonuclear neutrophil (maximum) |
| Gestational age at study entry | Bands |
| Corrected gestational age at study entry | Platelets (minimum and maximum) |
| Weight at study entry | Blood glucose (maximum) |
| Number of days in the NICU | C-reactive protein (maximum) |
| Vasopressor use | Venous lactate (maximum) |
| Intubation | Arterial lactate (maximum) |
| Number of days on a ventilator (total) | Blood urea nitrogen (maximum) |
| Number of days on a ventilator at study entry | Serum Creatinine (maximum) |
| Umbilical venous catheter placement, duration < 1 week | Alanine aminotransferase (maximum) |
| Umbilical venous catheter placement, duration > 1 week | Aspartate aminotransferase (maximum) |
| Total duration of umbilical venous catheter placement | Alkaline phosphatase (maximum) |
| Duration of umbilical venous catheter placement at study entry | Bilirubin (maximum) |
| Mortality possibly related to bacteremia | International Normalized Ratio (maximum) |
| Survival at end of NICU admission | Prothrombin time (maximum) |
| Early antibiotic use (within the first 48 hours of life) | Albumin (minimum) |
| Mean arterial pressure (minimum and maximum) |  |
| Heart rate (minimum and maximum) |  |
| Temperature (minimum and maximum) |  |
| Respiratory rate (minimum and maximum) |  |
| Fraction of Inspired Oxygen (minimum and maximum) |  |
| Lethargy |  |
| Apnea |  |
| Temperature instability |  |
| Heart rhythm instability |  |
| Pale/grey skin colour |  |
| Mottled skin |  |
| Petechial rash |  |
| Abdominal distension |  |
| Poor peripheral perfusion |  |
| Younger maternal age |  |
| Premature rupture of membranes (maternal) |  |
| Intra-amniotic infection (maternal) |  |
| Corticosteroid use (maternal) |  |

**Table S2. Patient Characteristics of Entire Study Population (N=153)**

| **Parameter** | **Cases^a^ (n=42)** | **Matched^a^ Controls (n=42)** | **P value*** | **OR** | **95% CI** | | |  | **Unmatched^a^ Controls (n=69)** | | | **P value**** | |
| --- | --- | --- | --- | --- | --- | --- | --- | --- | --- | --- | --- | --- | --- |
| Gender *(% male)* | 57 | 52 | 0.8267 | 1.212 | 0.5 | - | 2.9 | | |  | 48 | | >0.05 |
| Gestational Age at Birth (weeks) | 26 (24-40) | 27 (24-39) | 0.3484 |  |  |  |  | | |  | 32 (24-42) | | <0.0001 |
| Days Post Birth at Study Entry | 10 (4-60) | 10 (0-60) | 0.5519 |  |  |  |  | | |  | 1 (0-26) | | <0.0001 |
| Length of Stay at Study Entry (days) | 9 (0-41) | 9 (0-59) | 0.6354 |  |  |  |  | | |  | 1 (0 – 26) | | <0.0001 |
| Corrected Gestational Age^b^ at Study Entry (weeks) | 28 (25-41) | 29 (25-39) | 0.4256 |  |  |  |  | | |  | 32 (27-42) | | <0.0001 |
| Weight at Study Entry (grams) | 870  (540-3118) | 978  (557-3300) | 0.0811 |  |  |  |  | | |  | 1615 (540-4025) | | <0.0001 |
| 48 hour Antibiotic Use^c^ *(% Yes)* | 95 | 90 | 0.6758 | 2.1 | 0.4 | - | 12.2 | | |  | 83 | | >0.05 |
| Total Length of Stay^d^ (days) | 38 (6-136) | 46 (8-115) | 0.4742 |  |  |  |  | | |  | 7 (1-71) | | <0.0001 |
| Survival at End of Stay (% Yes) | 93 | 100 | 0.2410 | 0.1 | 0.01 | - | 2.7 | | |  | 99 | | >0.05 |
| ^a^median (Range), unless otherwise indicated  ^b^Corrected Gestational Age (in weeks; equal to GA plus the number of days post-birth at time of study entry)  ^c^Percentage of neonates exposed to antibiotics for 48 hours in the immediate post-natal period.  ^d^Refers to length of stay in the level 3 NICU; majority of babies are transferred to a level 2 NICU once clinically stable.  *Statistical Test Used: For interval data: Parametric data with equal standard deviations, unpaired t-test; Parametric data with unequal standard deviations, unpaired t-test with Welch correction; Non-parametric data, Mann Whitney test. For nominal data: Fisher’s exact.  **Statistical Test Used: For interval data: Parametric data with equal standard deviations, ANOVA with Tukey's Post Test; Parametric data with unequal standard deviations or non-parametric data, Kruskal Wallis with Dunn's Post Test; For nominal data: Chi square for goodness of fit with Marascuillo Post Test. | | | | | | | | | | | | | |

| **Table S3. Characteristics of Patients Included in Final Bacteremia Tool** | | | | | | | | | | | | | |
| --- | --- | --- | --- | --- | --- | --- | --- | --- | --- | --- | --- | --- | --- |
| **Parameter** |  | **Cases^a^ (n=31)** |  |  |  |  | **Controls^a^ (n=30)** |  |  |  | **P-value^b^** | **Odds Ratio** | **95% Confidence Interval** |
| **Gender – Males** |  | 18 | ( | 58% | ) |  | 18 | ( | 60% | ) | >0.999 | 1.1 | 0.4 - 3.0 |
| **Gestational Age at Birth (Weeks)** |  | 28 | ± | 3 |  |  | 32 | ± | 4 |  | **<0.0001** |  |  |
| *(Range)* | ( | 24 | - | 40 | ) | ( | 27 | - | 40 | ) |  |  |  |
| **Days Post-Birth at Entry** |  | 9 |  |  |  |  | 1 |  |  |  | **<0.0001** |  |  |
| *(Range)* | ( | 4 | - | 41 | ) | ( | 0 | - | 7 | ) |  |  |  |
| **Length of Stay at Study Entry (days)** |  | 8 |  |  |  |  | 1 |  |  |  | **<0.0001** |  |  |
| *(Range)* | ( | 0 | - | 40 | ) | ( | 0 | - | 7 | ) |  |  |  |
| **Corrected GA at Study Entry (Weeks)** |  | 29 | ± | 3 |  |  | 32 | ± | 3 |  | **0.0008** |  |  |
| *(Range)* | ( | 25 | - | 41 | ) | ( | 28 | - | 40 | ) |  |  |  |
| **Weight at Study Entry (g)** |  | 873 |  |  |  |  | 1444 |  |  |  | **<0.0001** |  |  |
| *(Range)* | ( | 540 | - | 3118 | ) | ( | 970 | - | 4025 | ) |  |  |  |
| **48 hour antibiotic use^c^ (% Yes)** |  | 29 | ( | 94% | ) |  | 24 | ( | 80% | ) | 0.1466 | 3.6 | 0.7 - 19.6 |
| **Total Length of Stay^d^** |  | 45 | ± | 33 |  |  | 13 | ± | 14 |  | **<0.0001** |  |  |
| *(Range)* | ( | 6 | - | 136 | ) | ( | 1 | - | 58 | ) |  |  |  |
| **Survival at end of stay** |  | 30 | ( | 97% | ) |  | 29 | ( | 97% | ) | >0.999 | 1.0 | 0.06 - 17.3 |

^a^Number (%) of patients; for parametric data – mean ± SD (for data that passed the Kolmogorov-Smirnov normality test), or median (for data that failed to pass the Kolmogorov-Smirnov normality test); (range).

^b^Fisher’s exact test for nominal data; Two-tailed unpaired t-test for normally distributed interval data with equal standard deviations; Two-tailed unpaired t-test with Welch correction for normally distributed interval data with unequal standard deviations; Mann-Whitney U test for interval data that were not normally distributed; Bolded values denote significant *p*-values.

^c^Number (%) of neonates exposed to antibiotics for 48 hours in the immediate post-natal period.

^d^Refers to length of stay in the level 3 NICU; majority of babies are transferred to a level 2 NICU once clinically stable.

**Table S4. Microbiological Characteristics in Blood Cultures**

|  | **Number of Isolates (n=45)** | **Percent of Isolates** |
| --- | --- | --- |
| **Gram Positive Bacteria** | **38** | **84** |
| Methicillin Susceptible *S. aureus* | 1 | 2.6 |
| Methicillin Resistant *S. aureus* | 0 | 0.0 |
| Coagulase Negative Staphylococci | 29 | 76.3 |
| Group B *Streptococcal spp.* | 5 | 13.2 |
| *Streptococcal spp.* other | 1 | 2.6 |
| *Enterococcus faecalis* | 2 | 5.3 |
| **Gram Negative Bacteria** | **7** | **16** |
| Enterobacteriaceae | 7 | 100.0 |
| *E. coli* Total | 7 | 100.0 |
| Non-Extended Spectrum β-lactamase producing *E. coli* | 6 | 85.7 |
| Extended Spectrum β-lactamase producing *E. coli* | 1 | 14.3 |
| **Concomitant Sources with Positive Cultures** | **18** | **Percent of Total Number of Positive Concomitant Sources** |
| Blood^a^ | 1 | 5.6 |
| Urine | 8 | 44.4 |
| Skin | 1 | 5.6 |
| Cerebrospinal fluid | 4 | 22.2 |
| UVC catheter tip | 3 | 16.7 |
| Peripheral IV tip | 1 | 5.6 |
| ^a^One patient had a second blood culture drawn later on the same day  UVC: Umbilical Venous Catheter; IV: Intravenous | | |

| **Table S5. Parameters Significantly Associated with Bacteremia (Univariate Analysis)** |
| --- |
| **Parameters Found to be Significantly Correlated with Bacteremia (Correlation Matrix) and Significantly Different Between Bacteremic and Non-Bacteremic Neonates by Univariate Analysis were:** |
| **Significant Correlation with Bacteremia at p<0.01:** |
| Number of days in the Neonatal Intensive Care Unit (NICU), gestational age at entry, gestational age at birth, corrected gestational age at entry, weight at entry, intubation, number of ventilation days, intra-amniotic infection, total duration of umbilical venous catheter placement at entry, minimum heart rate, maximum heart rate, maximum fraction of inspired oxygen (FiO_2_), lethargy, apnea, temperature instability, pale/grey colour, mottled skin, abdominal distension, maximum white blood cell count, maximum polymorphonuclear neutrophil count, bands, maximum blood glucose |
| **Significant Correlation with Bacteremia at p<0.05:** |
| Mortality possibly correlated with bacteremia^a^, survival at the end of NICU stay, maximum mean arterial pressure, maximum temperature |
| ^a^Defined as death within 2 weeks of diagnosis of bacteremia.  *Note:* All variables were available for ≥20% of patients in infection or control group*.* |

| **Table S6. What Would Happen with Bacteremia Tool if Pre-Test Probability were Different?** | | | | |
| --- | --- | --- | --- | --- |
| **Pre-test Probability (Period Prevalence)** | **Positive Predictive Value** | **Negative Predictive Value** | **Negative Post-test Probability** |  |
| 1% | 4% | 100% | 0.1% |  |
| 2% | 8% | 100% | 0.2% | Study Hospital Period Prevalence |
| 3% | 10% | 100% | 0.3% |  |
| 3% | 12% | 100% | 0.4% |  |
| 4% | 16% | 99% | 1% |  |
| 5% | 19% | 99% | 1% |  |
| 10% | 33% | 99% | 1% |  |
| 15% | 44% | 98% | 2% |  |
| 17% | 48% | 98% | 2% | Period Prevalence from Okascharoen 2005 <9> |
| 20% | 53% | 97% | 3% |  |
| 25% | 60% | 96% | 4% |  |
| 27% | 63% | 96% | 4% | Period Prevalence from Kudawla 2008 <11> and Bekhof 2013 <13> |
| 29% | 65% | 95% | 5% | Period Prevalence from Singh 2003 <8> |
| 30% | 66% | 95% | 5% |  |
| 33% | 69% | 94% | 6% | Period Prevalence from Okascharoen 2007 <6> |
| 35% | 71% | 94% | 6% |  |
| 39% | 74% | 93% | 7% | Period Prevalence from Dalig 2006 <10> |
| 40% | 75% | 93% | 7% |  |
| 41% | 76% | 92% | 8% | Period Prevalence from Mahieu 2000 <7> |
| 45% | 79% | 91% | 9% |  |
| 50% | 82% | 89% | 11% |  |
| 51% | 82% | 89% | 11% | Screening Tool Development Cohort Period Prevalence |
| 54% | 84% | 88% | 12% | Period Prevalence from Rosenberg 2010 <12> |
| 55% | 85% | 87% | 13% | Period Prevalence from Mahieu 2002 <5> |
| 56% | 85% | 87% | 13% |  |
| 60% | 87% | 85% | 15% |  |
| 70% | 91% | 78% | 22% |  |
| 80% | 95% | 67% | 33% |  |
| 90% | 98% | 48% | 52% |  |
| 100% | 100% | 0% | 100% |  |

**Table S7. Patient Characteristics of Validation Cohort (N=8)**

| **Parameter** | **Cases^a^ (n=7)** | **Controls (n=1)** |
| --- | --- | --- |
| Gender (% male) | 6 (86) | 1 (100) |
| Gestational Age at Birth (weeks) | 29 (26-31) | 33 |
| Days Post Birth at Study Entry | 9 (0-12) | 0 |
| Length of Stay at Study Entry (days) | 9 (0-12) | 0 |
| Corrected Gestational Age^b^ at Study Entry (weeks) | 29 (27-32) | 33 |
| Weight at Study Entry (grams) | 1212 (769-1351) | 1770 |
| 48 hour Antibiotic Use^c^ (% Yes) | 7 (100) | 0 (0) |
| Total Length of Stay (days) | 30 (13-128) | 12 |
| Survival at End of Stay (% Yes) | 7 (100) | 1 (100) |
| ^a^median (Range), unless otherwise indicated  ^b^Corrected Gestational Age (in weeks; equal to GA plus the number of days post-birth at time of study entry)  ^c^Percentage of neonates exposed to antibiotics for 48 hours in the immediate post-natal period. | | |
