# Supplementary figures and images for: Development, evaluation and validation of a screening tool for late onset bacteremia in neonates – a pilot study
Source: BMC Pediatr. 2019 Jul 24;19:253. doi: 10.1186/s12887-019-1633-1 (PMC6651932; doi:10.1186/s12887-019-1633-1)

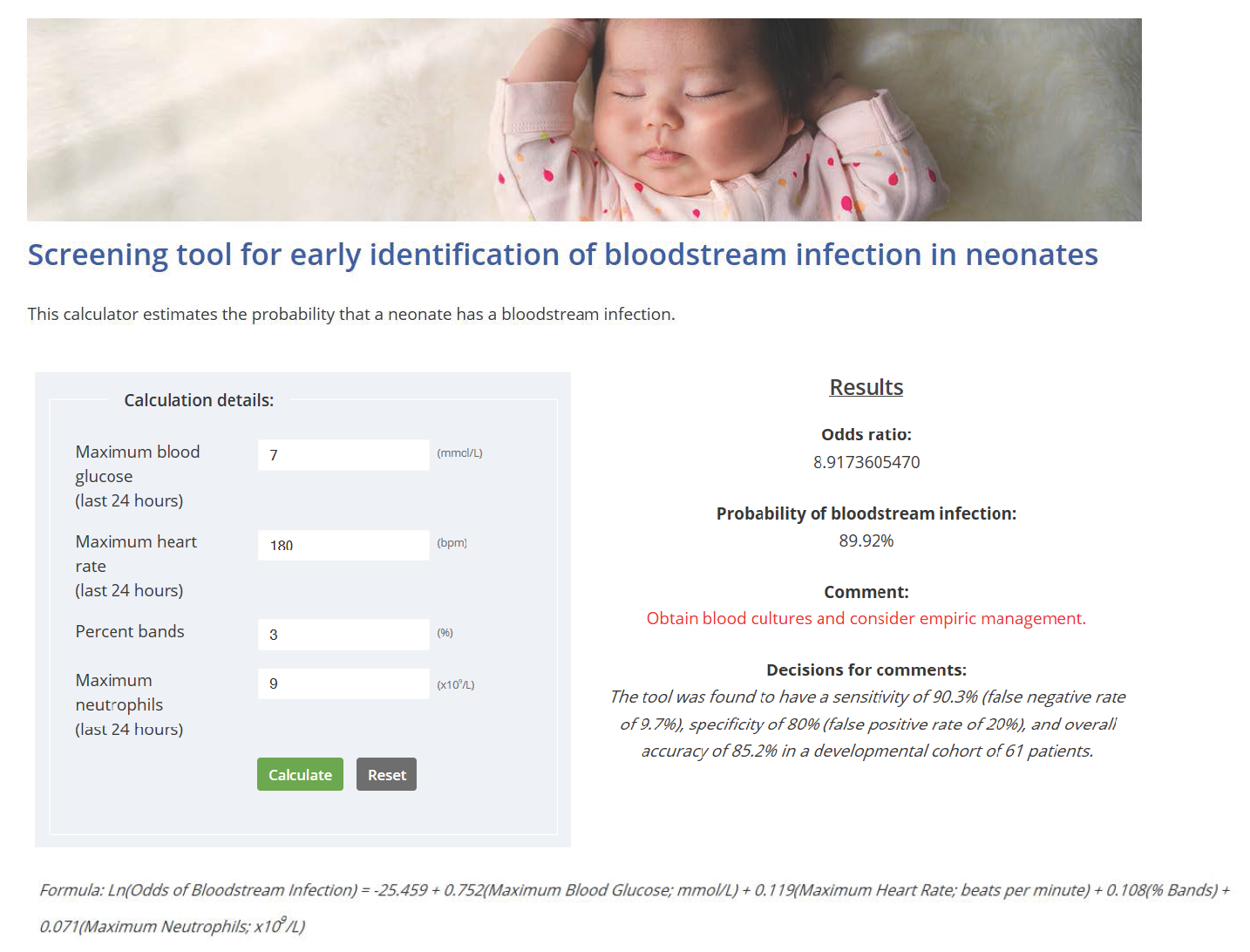

Supplement: Supplementary file 2 — : Figure S1. Screening tool for early identification of bloodstream infection in neonates. This figure provides a screenshot of the screening tool. (TIF 887 kb) [file 12887_2019_1633_MOESM2_ESM.tif]
